# Quantitative Risk Assessment of Foot-and-Mouth Disease (FMD) Virus Introduction Into the FMD-Free Zone Without Vaccination of Argentina Through Legal and Illegal Trade of Bone-in Beef and Unvaccinated Susceptible Species

**DOI:** 10.3389/fvets.2019.00078

**Published:** 2019-03-19

**Authors:** Andrea Marcos, Andrés M. Perez

**Affiliations:** ^1^Epidemiology Coordination, Animal Health National Directorate, National Service of Animal Health and Agrifood, Buenos Aires, Argentina; ^2^Department of Veterinary Population Medicine, Center for Animal Health and Food Safety, College of Veterinary Medicine, University of Minnesota, St Paul, MN, United States

**Keywords:** foot and mouth disease, quantitative risk assessment, beef, swine, sheep, Argentina

## Abstract

Argentina is a foot-and-mouth disease (FMD)-free country divided into five zones associated to disparate epidemiological situations and control strategies. Two zones are free from FMD with vaccination and three without vaccination. Quantitative risk assessment was used here to estimate the risk of introduction of FMD virus (FMDV) into the Argentine FMD-free without vaccination zone via legal or illegal trade of bone-in beef and non-vaccinated live animals from the FMD-free zone with vaccination of the country. Because trade of those commodities between those two zones is currently banned in Argentina, the analysis here will help evaluating the impact of relaxing such prohibition in the national regulation and the impact of illegal trade. Results suggest that if the volume of incoming bone-in beef is equal to the volume of deboned beef that enters the non-vaccinating zone, the annual risk of an FMDV introduction to the zone without vaccination will be low (0.0017). Indeed, the risk of introduction per kg trade volume via illegal trade is 6.9 times higher compared to legal trade. Similarly, the annual risk was also low for movement of live sheep and goat (0.0059) and swine (0.007) when the FMDV was assumed to be adapted to bovine and when a serological test was performed prior to movement. The implementation of a serological test to sheep and goat reduces 19 times the risk for FMDV introduction. In conclusion, the risk of introduction of FMDV into the FMD-free zone without vaccination through bone-in beef, sheep, goat, and swine with certain requirements, such as serological testing, is nil. If legal trade was allowed, the incoming risk may even be lower, compared to the current scenario of prohibiting the introduction. Results are likely due to the controls associated to legal trade, and the subsequent reduction of illegal trade. Consequently, results suggest that a policy of incentive and facilitation of good practices may be more effective in preventing FMDV introduction into a free zone than prohibition of trade.

## Introduction

Foot and Mouth Disease (FMD) is considered one of the most important diseases affecting animals due to its high transmissibility and associated productive and economic losses. The FMD virus (FMDV), which was the first animal virus to be identified (1), belongs to the Aphthovirus genus of the Picornaviridae family. Seven distinct serotypes have been identified (A, O, C, SAT1, SAT2, SAT 3, and Asia 1), without cross-immunity among them (2). The FMDV survives cold temperatures but it is inactivated at 70°C for 30 min and it is sensitive to low pH values (inactivated in a few hours at pH 6, and few seconds at pH 5) (3). For that reason, the muscle lactic acidification process that occurs during the “rigor mortis” phase of meat processing is considered effective for virus elimination in infected animals. Nevertheless, the FMDV may survive in lymph nodes and bone marrow where pH does not decrease as much as in muscle (4).

Cattle are the primary host for the FMDV, although the virus may affect all cloven-hoofed animals (pigs, sheep, goats, and certain wildlife species) (5). The disease typically courses with a high morbidity rate, close to 100%, except for the SAT serotypes whose morbidity rates is near 12–15% (6). The mortality rate is low (1–5%) in adult animals, that may increase up to 20% in calves (7). Common signs of infection are depression, weakness, fever, anorexia, tremors, decreased milk production, salivation, bruxism, ambulatory problems, and vesicles filled with clear fluid in mouth, tongue, lips, gums and palate mucosa, skin, interdigital space, and coronet, that may also appear on the teats and udder (8). The severity of clinical signs varies with a number of epidemiological factors, including strain virulence, infective dose, animal age and breed, and host immunity (7).

The FMDV incubation period in cattle varies from 3 to 10 days depending on the viral strain, the type of exposure, and the host immune status (4). According to the Terrestrial Animal Health Code of the World Organization for Animal Health (OIE), the incubation period is 14 days. Transmission usually occurs through inhalation of aerosolized virus or by direct contact with infected animals. Transmission through skin or mucous membranes wounds is not efficient. Calves may be orally infected by milk containing FMDVs. The initial site of virus replication is the pharyngeal area. Due to the subsequent viremia, the FMDV arrives to hoof and oral epithelia. When antibodies are produced, the virus disappears from blood. Infected animals start spreading virus 1 week before the onset of clinical signs, and 1 to 7 days post-symptoms, spread of viral particles is typically evident (4). The disease condition is typically resolved in 8 to 15 days.

In pigs, oral infection is the main route of infection, and, comparing with cattle and sheep, they are more resistant to airborne infection (9), even though pigs excrete by respiratory route more virus than cattle. Pigs do not serve as disease carriers, but they may act as multiplier agents (10). When pigs are exposed to low doses of virus they may develop a sub-clinical or a mild form of the disease without efficient transmission (11). The incubation period may be 2 days or longer. Some strains of the O Taiwan 1997 FMDV are highly virulent for swine and in those cases, clinical signs may appear 18 h post-exposure (11).

Clinical detection is difficult in sheep; hence, they are believed to play an important role in disease transmission because infection spreads unnoticed. According to experimental studies, 21–27% of infected sheep do not develop signs and 20% show only a single lesion (12, 13). However, they excrete virus by the respiratory system. Sheep are highly susceptible to airborne infection, though less than cattle because of their relatively small lung capacity. Transmission between sheep is believed to be relatively ineffective and contact with other species may be required to maintain and spread the infection (14). Sheep may, however, become virus carriers (15).

**Figure 1:**
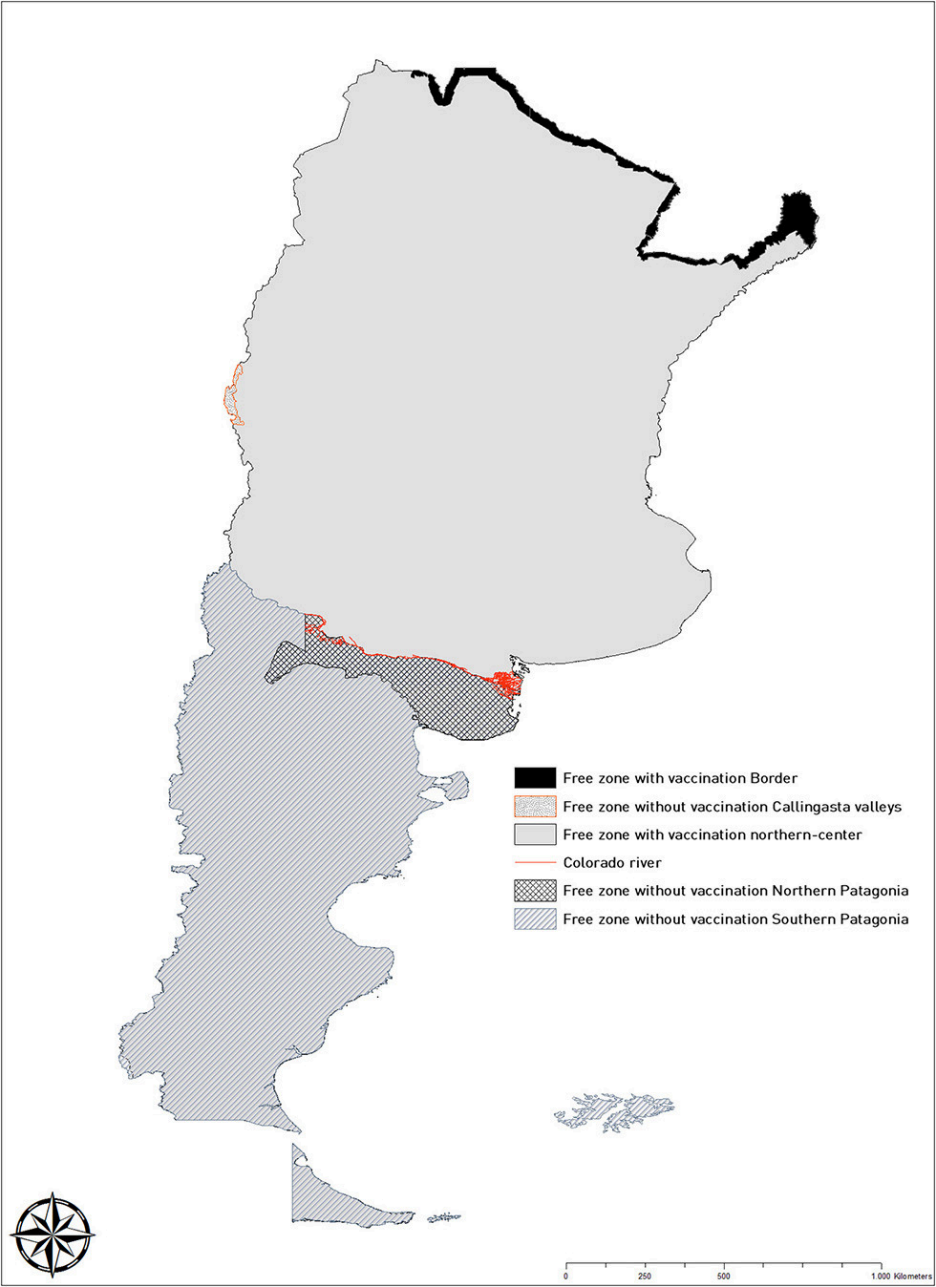
Zonification of Argentina (2018) according to foot-and-mouth disease status into three zones without vaccination and two zones with vaccination. The Colorado River, which serves as a major ecological barrier in the country, is indicated.

In Argentina, two major ecosystems are distinguished for FMD, namely, the endemic and free natural ecosystems. The endemic ecosystem, northern to the Colorado River, includes some of the most intensive and densely populated areas of the country, whereas in the free natural ecosystem, southern to the Colorado River and generally referred to as the Patagonia region, production is extensive and with very low contact rates. By the time when this article was written in December 2017, Argentina had FMD-free status, granted by OIE. The country was divided into five zones, associated to disparate epidemiological disease situations and implementation of differential control strategies. Those five zones included: (1) the free zone with vaccination that covers most of the territory northern to the Colorado River; (2) the free zone with vaccination at the northern border, where intensive surveillance activities are conducted; (3) and three free zones without vaccination (Callingasta valley, which is an isolated and relatively small zone in the Andeans, and two zones located southern to the Colorado River, referred to as Northern and Southern Patagonia, respectively) (Figure 1).

According to the OIE, to maintain the FMD-free status for international trade, it is necessary to demonstrate the absence of clinical signs of disease, through active surveillance for early detection of the disease. Additionally, the absence of FMDV infection has to been proved. To avoid the introduction of the FMDV, countries reduce or restrict trading of animals and by-products. Likewise, in Argentina, trade of certain products has traditionally been restricted between zones with different disease status. OIE standards specify that, for fresh beef imports from countries or zones free from FMD with vaccination, it is only necessary to ensure that the animal has truly originated from the FMD-free zone with vaccination (FZWV), and that no FMD-like signs were detected at the ante- and post-mortem inspection. Regarding movement of live ruminants and domestic pigs from FMD-FZWV into FMD-free zones without vaccination (FZWoutV), OIE standards require ensuring that the animals have not shown any clinical sign of FMD on the day of shipment. Also, the animals must remain in a country or FMD-free zone since birth, or for at least the last 3 months, and if their destination is an FMD-FZWoutV, they should not be vaccinated and should be negative in tests for the detection of FMDV antibodies (16).

Current legislation in Argentina is stricter compared to international regulations, it establishes that only matured and deboned beef may enter into the FMD-FZWoutV. That legislation has been informed by scientific studies that suggest that the FMDV may survive in bone marrow up to 7 months in frozen beef (17). Introduction of FMD-susceptible live animals into the FMD-FZWoutV is also banned because susceptible unvaccinated animals may be asymptomatic carriers of the virus (18). However, the carrier state has only been described in cattle, sheep, and goat, and it has never been shown that they could actually generate a disease outbreak under field conditions (14).

Constraints generated by those regulations, along with climatic factors, such as prolonged drought, may result in shortages of certain products, such as short ribs, in the FMD-FZWoutV.

For breeding swine, sheep and goat, inability to move live animals into FMD-FZWoutV impairs the potential for genetic improvement of the livestock production in the region. That situation affects the social development of the country, because, given that the region has the status of FMD-FZWoutV, it has potential for providing quality products to the international market, which cannot be fully achieved because of the limitations in genetic improvement of its livestock.

Consequently, one may argue that the current legislation, initially intended to protect the resources and status of FMD-FZWoutV, is unnecessarily limiting the production, economic, and social development of the region.

The objective of the study was to quantitatively assess the risk of introduction of FMD through susceptible unvaccinated animals and bone-in beef from the FMD-FZWV into the FMD-FZWoutV of Argentina. We focused only on those routes because those are the relevant pathways to be assessed when considering the possibility for modifying the national legislation. Cattle movement have not been considered because according OIE standards to remain as a FMD free country or zone where vaccination is not practiced, introduction of vaccinated animals must be banned. Eventually, results here will help to evaluate the legislation associated with FMD control in Argentina, improve production conditions, and facilitate internal trade while complying with the highest safety standards required by international markets.

## Materials and Methods

### Analytical Approach

An analytical methodology, referred to as quantitative stochastic risk assessment, similar to those used for FMD risk assessments conducted in the U.S. and Spain were used here (19, 20). Briefly, all steps needed for occurrence of the event of interest were plotted in scenario trees. For each step or node in the scenario tree, a probability distribution was assumed based on information available from the literature and the data sources described here. For all the probabilities, the time frame assessed was the period before detection of the epidemic, which is sometimes referred as the silent phase of an epidemic.

Two models were run, a model for assessing the risk associated with trade of bone-in beef and a model for assess the risk associated with animal movements.

Analyses were conducted running 10,000 simulations implemented in the @ Risk version 5.5.1 software (Palisade Corporation, 2010. Ithaca, NY, USA). Sensitivity of the results to the model parameterization was assessed, for each scenario, by measuring Spearman's rank correlation between the model output (i.e., the predicted risk) and the model parameters.

### Data Sources and Assumptions

#### Time Period

**Figure 2:**
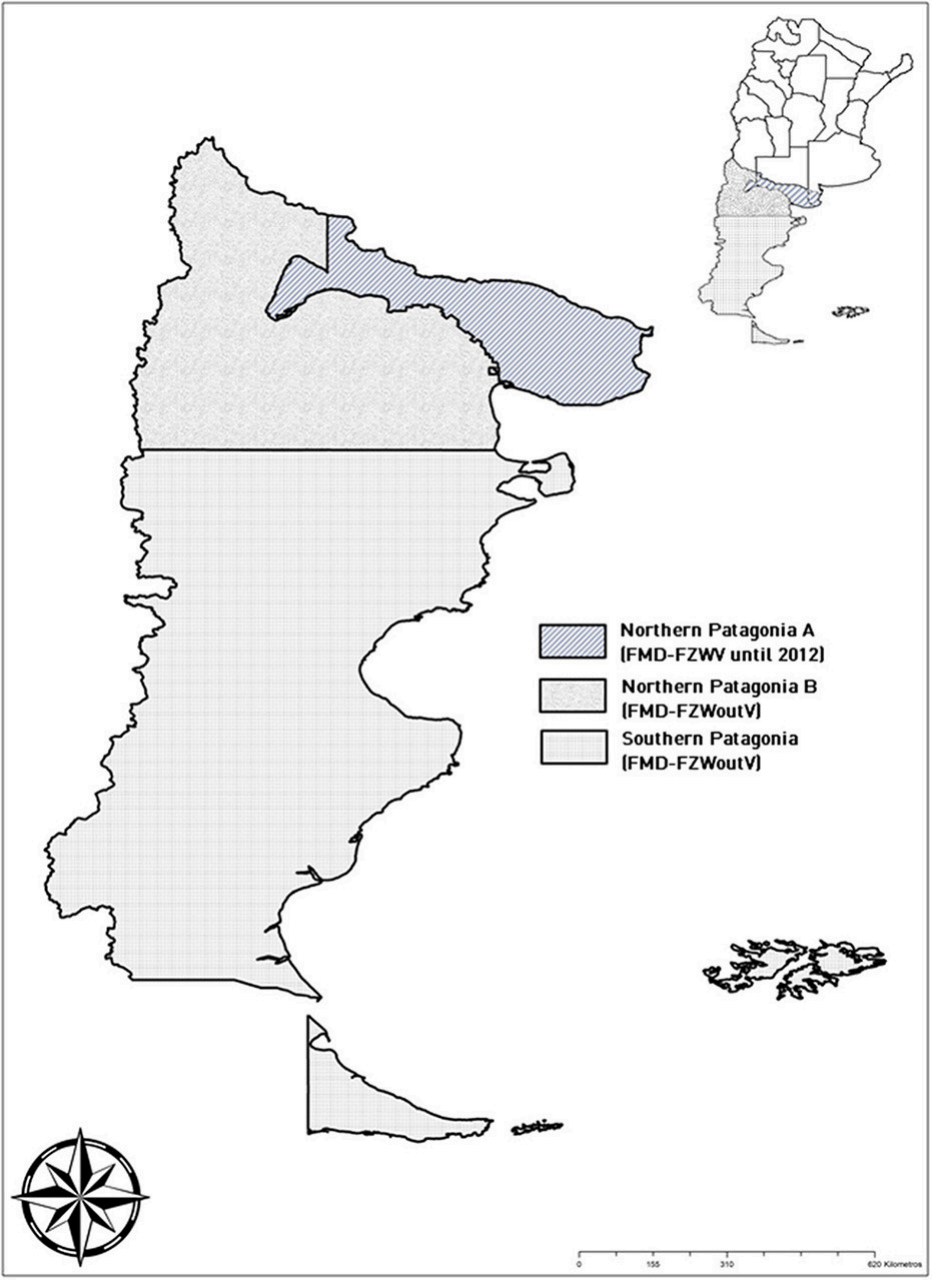
Zonification of Patagonia until 2012.

Data used for this quantitative assessment related to the time period from 2002 through 2010, when Northern Patagonia was divided into two zones referred to as Northern Patagonia A, with FMD vaccination, and Northern Patagonia B and Southern Patagonia, without FMD vaccination (Figure 2). At that time, introduction of bone-in beef incoming from the FMD-FZWV was allowed into Northern Patagonia A, but not into the rest of Patagonia.

#### FMD Data

Argentina is free from FMD since 2006. The OIE official FMD-FZWV status was regained in May 2007, except for the high surveillance zone that recovers the status in February 2011. All the scenarios assessed here are hypothetical because the trade of bone-in beef and non-vaccinated live animals between zones is banned. The surveillance program has not detected any viral circulation in FMD-FZWV and for that reason, the risk for introduction into the FMD-FZWoutV may be considered nil. However, there is certainly a risk that FMDV introduction into the FMD-FZWV may occur and remain unnoticed for a certain period of time and that during that silent phase of a hypothetical epidemic, infected animals or products may be introduced into the FMD-FZWoutV. For that reason, to evaluate the risk of introduction into the FMD-FZWoutV, the worst-case scenario of an FMD outbreak occurring in the FMD-FZWV of Argentina was assumed here. Risk was assessed considering the silent phase of the epidemic, because once the National Animal Health and Agrifood Quality Service (SENASA) detects FMD-like clinical signs or serological positivity, all movements of animals, and animal products would be banned. Data related to FMD risk used for calculating the probability that one premises in the FMD-FZWV was FMDV-infected (P1a) and the probability that at least one animal was infected and not detected with FMDV (P2a) were obtained from the database of SENASA and collected from FMD outbreaks reported in Argentina in 2000, 2001, 2002, 2003, and 2006. For each reported outbreak (*n* = 2565), data available to us included likely start date based on age of lesions, official intervention date, size of susceptible population, and number of sick animals.

#### Animal and Beef Data

The volume of traded beef annually entering into the Patagonian region was estimated using data provided by the Patagonic Phytozoosanitary Barrier Foundation (FUNBAPA) and collected in 2002–2010. Data on movements of cattle into slaughterhouses, collected on a daily basis through 2010, were obtained from the SENASA Integrated Management System for Animal Health (SIGSA) and Health Management System (SGS). Movements of pigs, sheep, and the same sources but are from 2011. To calculate the probability that FMDV-infection was not detected at the ante-mortem (P4a) and post-mortem (P5a) inspections, the estimation of vaccinated animals with partial immunity which could experience subclinical disease and spread viruses without detectable signs was based on the results of the annual surveillance activities. At the time of the analysis, results were available from 2003 to 2008.

#### Acceptable Risk

The definition of risk varies depending on the context in which the term is used. Here, we use the term risk to refer to the probability of introduction of FMD virus into the FMD-FZWoutV of Argentina from the FMD-FZWV through bone-in beef and non-vaccinated live animals. In consultation with SENASA officers, the maximum level of risk acceptable was set up on an average of 0.01. Any estimated risk below this value would be considered negligible. The maximum level of acceptable risk is always subjective, and related to economic, social, and political considerations of a country. At the time when this manuscript was written in 2017, Argentina has not suffered an FMD outbreak in the FMD-FZWV for more than 10 years. Thus, assuming a frequency of epidemics in the FMD-FZWV of once every 10 years, if, for any of those epidemics the risk for spread into the FMD-FZWoutV was 0.01, then it would imply that, on average, one would expect the event to occur once every 1000 years, which was considered negligible.

### Model Formulation and Definition of Distributions of Input Variables

#### Model Formulation for Assessment of Risk Associated With Trade of Bone-in Beef (Figure 3)

This model estimates the risk of FMD virus enters to the FMD-FZWoutV through legally and illegally traded beef from the FMD-FZWV. The relation between the vaccinated bovine immunity level and the presence of the FMDV in bone marrow is considered.

**Figure 3:**
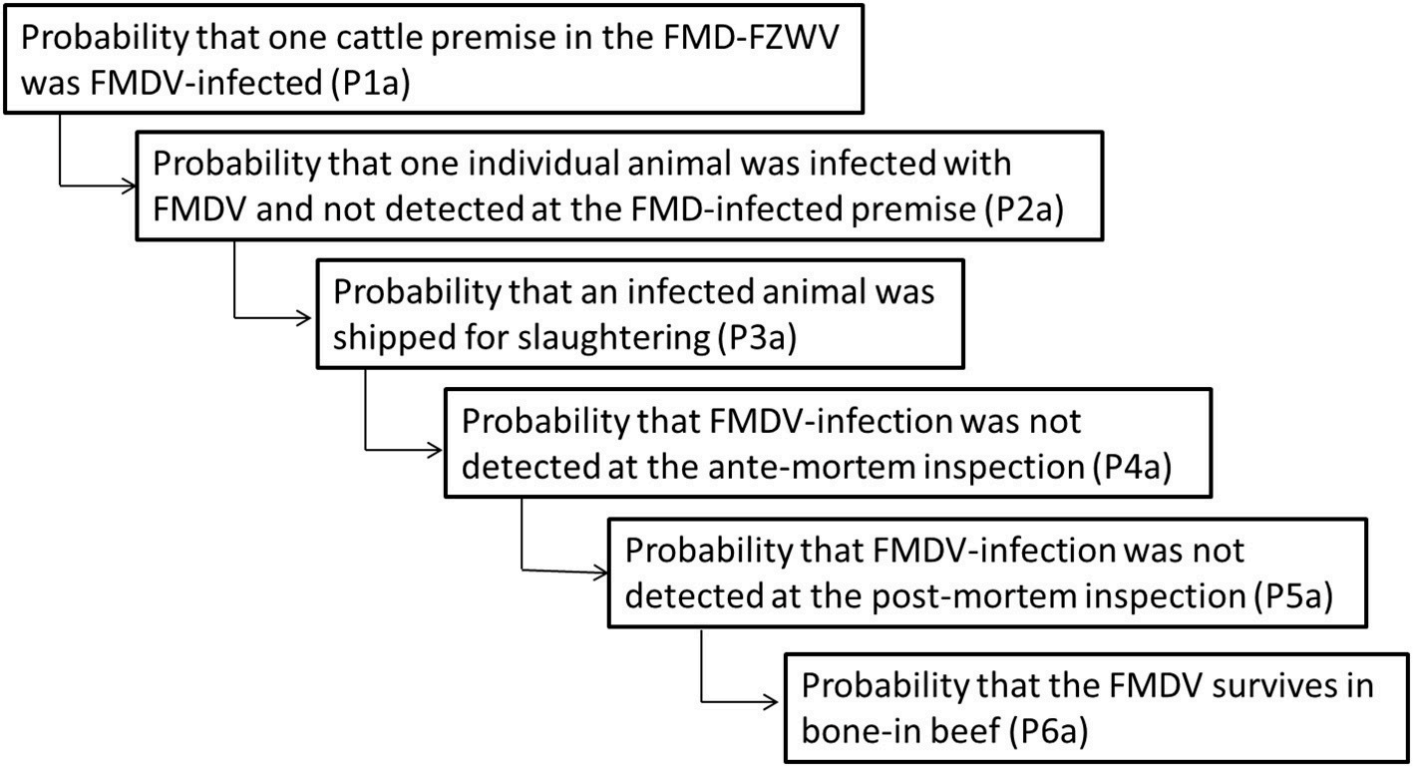
Representation of the model input parameters and the sequence of events for an assessment of the risk associated with trade of bone-in beef.

##### Volume of traded beef (n)

For the computation of the overall probability that FMDV-infected bone-in beef reaching FMD-FZWoutV, we estimated the amount of bone-in beef that would enter annually, if their trade was allowed following **two** alternative procedures and using data provided by FUNBAPA and collected in 2002–2010.

*Estimation 1: according to the ratio bone-in to deboned beef entering Northern Patagonia A until 2012*. The amount of bone-in and deboned beef annually entering areas Northern Patagonia A, Northern Patagonia B, and Southern Patagonia was quantified. Until 2012 bone-in beef were allowed to enter into Northern Patagonia A, because the region was assumed to be FMD-FZWV (Figure 2). We calculated the average bone-in beef/deboned beef ratio (1.32) that entered into Northern Patagonia A and assumed that the deboned beef required for Northern Patagonia B would equal such ratio. Total required bone-in beef on average would be of 39,115,635.58 kg/year, accordingly to the annual average amount of incoming deboned beef (29,633,057.26 kg/year). Because the average weight of a side of beef is about 84 kilos, 232,831 cattle should have been slaughtered to meet that demand.

According to a study conducted by an Argentina-U.S. joint committee in 1966 (21), repeated vaccinations reduce the risk for FMDV-infection in lymph nodes. Others found that vaccination prevents both viremia and vesicular lesions (22) and no viremia was detected in vaccinated cattle (23). Based on those data, risk here is likely associated to vaccinated animals with a partial level of immunity, which could be infected with FMDV and become a source of infection to in-contact susceptible species (12) but show no signs of disease, impairing chances of ante and post-mortem detection.

Immunity of vaccinated animals has annually been assessed in Argentina since 2003. Animal categories suitable for slaughtering have immunity levels (defined as the proportion of protected animals) that are at least 75% and typically >80% in Argentina, which is considered an excellent level of protection (24, 25). Consistently with the model of a worst-case scenario, we assumed that 75% of the cattle was protected, i.e., that 25% of the cattle did not have an adequate level of protection (partial immunity).

Given the assumption that 25% of the animals would have partial immunity, the total number of slaughtered bovines with partial immunity that could have a subclinical disease (n1) would be 56,200.

*Estimation 2: Considering illegal trade of bone-in beef*. The figure here was based on data from confiscation in different checkpoints between the FMD-FZWV and FMD-FZWoutV (FUNBAPA). During 2010, 5,246 kg of bone-in beef were confiscated. According to the inspectors of FUNBAPA, assuming that only 50% of the illegally traded bone-in beef is detected is a conservative assumption and the true value of detection may be higher than the 50%. All the analysis is based in assuming the “worst case scenario” for each probability and that is why we refer to some values as conservative. Thus, we may assume a value of illegal trade detection of 50% and therefore 5,246 kilos of beef with bone per year would enter the FMD-FZWoutV, i.e., the beef not confiscated by the checkpoints. Following the computation described above, that figure would be equivalent to 90 cattle (n2). However, because this scenario is associated with illegal trade, inspection may not have been conducted here and, thus the probability of detection at ante- and post-mortem inspections was considered impossible, i.e., 0%.

##### Probability that one cattle premise in the FMD-FZWV was FMDV-infected (P1a)

This is the probability of randomly selecting an FMDV-infected cattle premise. Some have considered the total number of outbreaks reported during an epidemic to model this probability (19). We considered, however, that such decision overestimates the risk and, for that reason, we considered only the number of outbreaks reported before detection of the epidemic (referred to as “silent phase”), as suggested elsewhere (20). Consequently, the number of premises with FMD outbreaks that started before the date of the first official intervention in 2000, 2001, 2002, 2003, and 2006 was considered to estimate P1a.

The starting date corresponded to the onset of signs, according to the farmer, or to the number of days when it was estimated the disease had begun, according to the analysis of the age of lesions observed by the SENASA veterinarians (7).

We estimated the probability that a farm had FMD but had not been detected by dividing the number of infected premises before the official SENASA intervention during 2000–2002, 2003, and 2006 outbreaks by the total number of cattle farms existing in 2002 in the FMD-FZWV, according the 2002 Argentinian agricultural census.

Based on those data, P1a was assumed to follow a Pert distribution with most likely value = 1.56 × 10^−5^, minimum value = 5.21 × 10^−6^, and maximum value = 3.12 × 10^−5^. Those values were extremely conservative. Note that for the last 10 years, SENASA consistently detected the first outbreak before a second outbreak actually occurred. For that reason, the most likely scenario is that future hypothetical epidemics in the FZWV would consist of one single outbreak. However, we preferred to maintain the most conservative values to be consistent with an analytical strategy of modeling the worst case scenario.

##### Probability that one individual animal was infected with FMDV and not detected at the FMD-infected premise (P2a)

This is the probability of choosing an FMDV-infected animal during the silent phase of an epidemic and given that the premise was FMDV-infected.

Within-herd prevalence was estimated in those premises in which the start date of the FMD outbreaks was prior to the first intervention of SENASA during the years 2000 and 2006. The probability that an animal was infected before the outbreak detection was computed by dividing the number of sick animals by the total population of the herd at the time of official intervention. This procedure led to estimates for P2a = 9% (CI95% = 1.4–25%).

Due to the small amount of information available the triangular distribution was the best fit to the data. The distribution was obtained transforming these calculated prevalences using log_10_. The values of the triangular distribution were: minimum: −1.88, media: −1.03, and maximum −0.6.

##### Probability that an infected animal was shipped for slaughtering (P3a)

Data from 427,000 movements into slaughter of all premises in Argentina in 2010 were analyzed, comparing the proportion of cattle each farm sent. The distribution that best fit the data was a lognormal distribution. Based on the data, the following values were set as the parameters for the lognormal distribution: mean 0.106 and standard deviation 0.189. Based, on those results, the minimum scenario was that <1% of the cattle in the farm were moved to slaughtering during the silent phase of an epidemic, with maximum being all the animals in the farms, and most likely value (median) being 5% of total animals in the premises.

##### Probability that FMDV-infection was not detected at the ante-mortem (P4a) and post-mortem (P5a) inspections

Because of the recent history of FMD outbreaks in Argentina, it is mandatory to screen for FMD-like clinical signs all animals at a slaughterhouse both before and after slaughtering. Ante-mortem inspection involves detection of febrile illness and injuries or signs such as salivation and impaired ambulation observing the whole shipment. Post-mortem inspection involves the detection of vesicular lesions in tongue, hoof and rumen. There are, however, FMD cases that may not show classic signs of infection. In those animals, the FMD may remain undetected during ante and post mortem inspection (26). Vaccinated animals with partial immunity may become animals with subclinical disease and may spread viruses without detectable signs of the disease (12, 26).

Based on immunity of vaccinated animals in Argentina we assumed that only 25% of the animals would be susceptible to the infection but the clinical disease would be masked and would remain undetected in the ante and postmortem inspection.

Assuming that post- mortem is five times more sensitive than ante-mortem inspection (27), and following values assumed by Abbiati et al. (28), we considered that P4a and P5a were Pert-distributed, with minimum, most likely, and maximum values of 0.5, 0.9, and 0.999 for P4a and 0.1, 0.18, and 0.1998 for P5a, respectively. Those values are equivalent to assuming with a 95%CI that 66–98% and 13–20% of infected animals would not be detected at ante-mortem and post- mortem inspection, respectively.

##### Probability that the FMDV survives in bone-in beef (P6a)

We assumed that if the animal was infected, the FMDV would certainly reach the bone. Therefore, the probability was one, which is a very conservative assumption, considering that those animals may have some level of immunity that could prevent viremia.

##### Risk Estimation

The probability of FMDV introduction to FMD-FZWoutV through bone-in beef from FMD-FZWV, P(I_bib_), would be estimated using the following formula:

(1) $$\begin{array}{l} \text{P}\left({\text{I}}_{\text{bib}}\right)=1-{\left(1-{\text{p}}_{\text{a}}\right)}^{\text{n}} \end{array}$$

(2) $$\begin{array}{l} {\text{p}}_{\text{a}}=\left(\text{P}1\text{a}\times \text{P}2\text{a}\times \text{P}3\text{a}\times \text{P}4\text{a}\times \text{P}5\text{a}\times \text{P}6\text{a}\right) \end{array}$$

#### Model Formulation for Assessment of Risk Associated With Animal Movements (Figure 4)

Studied species were sheep, goat, and swine. Sheep and goat populations were studied together because of the similar model parameterization. For pigs, two scenarios were assessed, namely, (1) FMDV bovine adapted strain, most common strains in the region, and (2) FMDV swine adapted strain, such as 1997 Taiwan strain (11). The swine strains have significant impact on swine populations, associated with high prevalence and more clinical symptoms, making easy to detect sick animals. For each species and scenario, the impact of performing, or not, a serological test for detection of FMD before shipping animals at the origin was assessed. The technique of choice was blocking ELISA liquid phase (LPB—ELISA), a sensitive, specific, and quantitative technique that allows for serotype identification, detecting antibodies to structural proteins.

**Figure 4:**
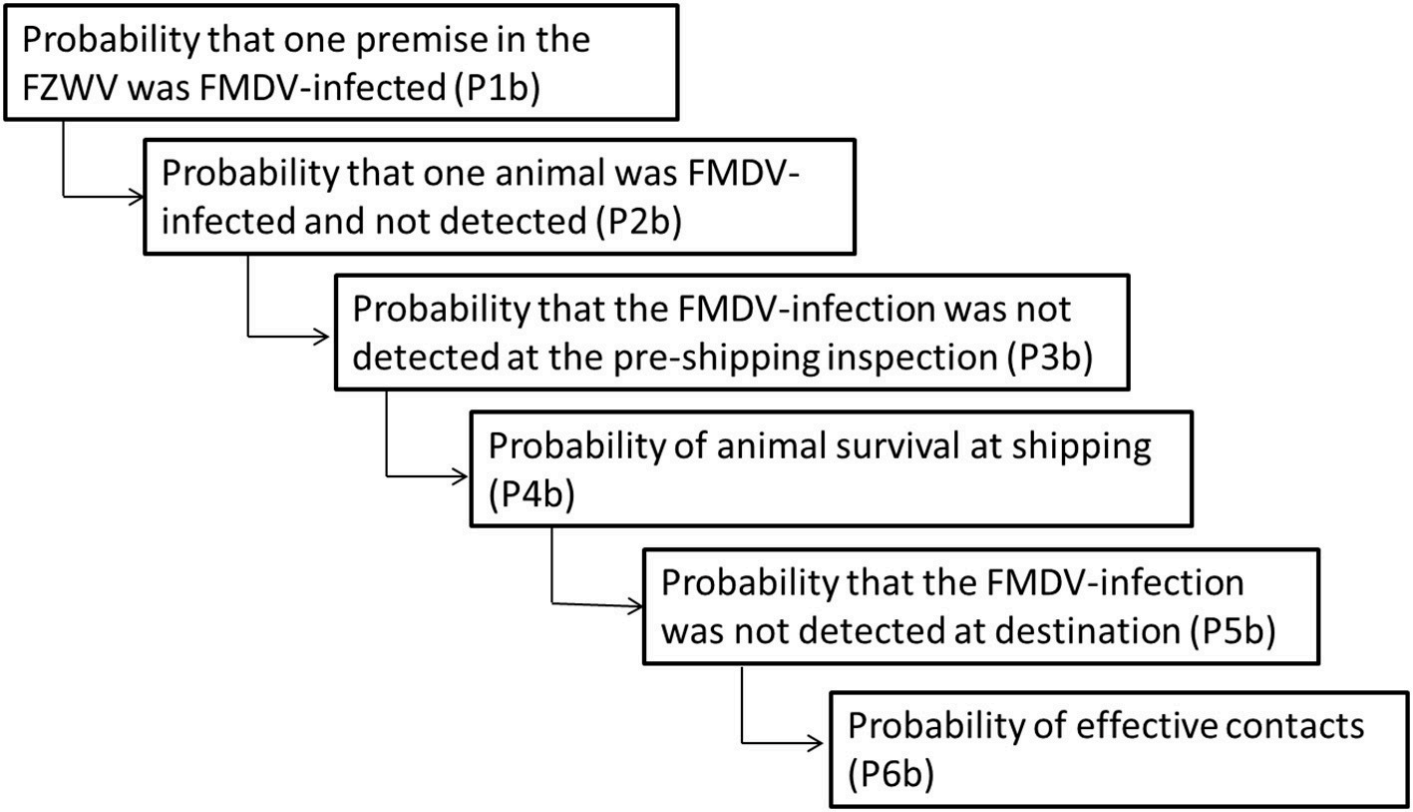
Representation of the model input parameters and the sequence of events for an assessment of the risk associated with animal movements.

##### Movements (n)

The animals expected to be moved into the FMD-FZWoutV would be only for breeding purposes. The number of animals was approximated using movement data from the country and certain assumptions. In 2011, 35,248 pigs were moved for breeding purposes in the country. The 99.5% of pigs' farms are in the FZWV. However, it is expected that if the incoming of breeding pigs were authorized, the interest of the industry would be important, due to access to international markets from a zone with FMD-FZWoutV status. Therefore, we assumed as a potential scenario that 10% of the movements will be redirected to FMD-FZWoutV (n3 = 3,500 pigs per year), although this is, likely, an overestimation, giving the few pig farms that currently exist in the area, that is consistent with the intention of assuming conservative values through the analysis here.

The situation is the opposite for sheep, because most of these animals are in FMD-FZWoutV. During 2011, 9.523 sheep and goat moved for breeding purposes into other farms in the FMD-FZWV. In the FMD-FZWoutV, 50,200 sheep were moved for breeding purposes. Therefore, if the possibility of shipping sheep and goat into the FMD-FZWoutV exists, the number of shipped animals would not be expected to be as large as for swine. Therefore, we assumed again that 10% of the movements will be redirected to FMD-FZWoutV, ~1.000 animals moved per year (n4).

##### Probability that one premise in the FZWV was FMDV-infected (P1b)

The parameter here was the probability of randomly selecting a pigs or sheep or goat premises infected with FMDV. The number of premises in which FMD-like signs was detected in pigs, sheep, and goat in 2000, 2001, 2002, 2003, and 2006 was considered, during the entire period of the epidemic. This is because there was no diseased pig or sheep and goat on the affected premises in Argentina prior to the detection of the epidemic, which, again, was a conservative assumption. We estimated the probability that premises had FMD by dividing the number of infected premises by the total pigs or sheep and goat premises that existed in Argentina in 2002.

Based on those data, P1b for the bovine adapted strain was assumed to follow a Pert distribution, for pigs most likely value = 1.6 × 10^−5^, minimum value = 1.6 × 10^−5^, and maximum value = 8.2 × 10^−4^; and for sheep and goat most likely value = 9.7 × 10^−6^, minimum value = 9.7 × 10^−6^ and maximum value = 1.2 × 10^−4^.

For the scenario involving a swine adapted strain, it was estimated that the between-herd prevalence would be four times higher than that observed in Argentina bovine adapted strains (29). Being that the between herd prevalence for the swine adapted strain was estimated to be four times higher than the bovine adapted strain, we multiplied by four the distribution estimated for the bovine adapted strain.

Those values were extremely conservative because, as has been previously explained, for the last 10 years SENASA consistently detected the first outbreak before a second outbreak actually occurred. However, we preferred to maintain the most conservative values to be consistent with an analytical strategy of modeling the worst-case scenario.

##### Probability that one animal was FMDV-infected and not detected (P2b)

We estimated the probability of an animal diseased calculating the ratio between the number of sick animals and the total population of the herd in all the premises affected in the moment of the first official intervention. With the data of all the premises we adjusted a Gauss inverted distribution with the following parameters (0.28649; 0.15036) for pigs.

The probability of choosing an FMDV-infected animal with the FMDV bovine adapted strain during an epidemic given that the premises was FMDV-infected was, on average, 28% (CI95% = 1–97%). For pigs with the FMDV swine adapted strain the within-prevalence is twice that the expected with a bovine adapted strain (29). This was computed by multiplying, on each simulation, the value withdrew from the distribution by two, and assuming a maximum value of 100% for P2b. This led to a right-skewed distribution, which, again, was a conservative assumption.

For sheep and goat the best fitting distribution was a lognormal (0.23829; 0.17698). With this distribution the P2b was, on average, 23% (CI95% = 5–74%).

##### Probability that the FMDV-infection was not detected at the pre-shipping inspection (P3b)

In Argentina official clinical inspection by SENASA veterinarian was mandatory for every animal before movement.

To estimate the P3b for pigs, data from the literature were obtained for ante-mortem (29) and clinical inspection in vaccinated pigs (30) to create a Pert distribution. We used as most likely value the probability of the infection not detected at ante-mortem inspection used by Lopez et al. (29), i.e., 80%. The minimum value, 50%, was taken from de Vos et al. (30) and is the minimum value of the Beta distribution used to estimate the sensitivity of clinical inspection during final screening after a one- and a 6-month waiting period if an area was declared free from FMD incorrectly. The maximum value was theoretical assuming the highest level of risk, with almost none animal detected (99%).

For the swine adapted strain we assumed that the detection would have double effectiveness and accordingly, the most likely value of 0.4, minimum of 0.25, and maximum of 0.495 were set. Given in Argentina, pigs are not vaccinated, it is expected that clinical signs will be more evident than in vaccinated animals, so values are considered conservative.

In the case of sheep and goat, based on data from the literature and considering that these species have less clinical symptoms (12, 13), another Pert distribution was assumed with most likely value = 0.95, minimum value = 0.75, and maximum value = 0.99.

The possibility of a serological test before the animal was shipped into its destination in the FZWoutV was then incorporated. In Argentina the validated test used in swine, sheep, and goat is the ELISA blocking liquid phase for the detection of structural proteins.

Sensitivity value estimation of the test is based on literature (31) and the probability of no detecting a FMDV-infected animal was assumed to follow a Pert distribution with the following values: most likely = 0.05 and 0, maximum = 0.31, and 0.31, minimum = 0 and 0 for pigs and sheep and goat, respectively.

##### Probability of animal survival at shipping (P4b)

It was considered that every animal would survive the shipping, which, again, is a conservative assumption.

##### Probability that the FMDV-infection was not detected at destination (P5b)

Because inspection at origin are conducted by SENASA officers, P5b was considered similar to P3b, although one may argue that P5b may be lower than P3b because of the higher opportunities for animals to develop and show clinical signs after the up-to-24 h transit. Thus, assuming that P3b = P5b is a conservative assumption.

##### Probability of effective contacts (P6b)

Because a quarantine period was not contemplated, we assumed that, if the animal was infected, then the contact with a susceptible animal at the farm of destination will certainly occur and will result in transmission of the virus and infection. This is a conservative assumption.

##### Risk estimation

The probability of FMDV introduction to FMD-FZWoutV through susceptible unvaccinated species from FMD-FZWV, P(I_sus_), would be estimated using the following formula:

(3) $$\begin{array}{l} \text{P}\left({\text{I}}_{\text{sus}}\right)=1-{\left(1-{\text{p}}_{\text{b}}\right)}^{\text{n}} \end{array}$$

(4) $$\begin{array}{l} {\text{p}}_{\text{b}}=\left(P1\text{b}\times \text{P}2\text{b}\times \text{P}3\text{b}\times \text{P}4\text{b}\times \text{P}5\text{b}\times \text{P}6\text{b}\right) \end{array}$$

## Results

Considering the assumed scenario of an undetected FMD epidemic in the FMD-FZWV, on average, once every 580 recurrences of FMD in Argentina the virus will reach the FZWoutV through bone-in beef, with a maximum (CI 95%) probability of once every 220 recurrences. The term recurrence here is used to refer to the introduction of the FMDV into the FMD-FZWV, which, by the time when this manuscript was written in 2017, has not occurred in Argentina for more than 10 years. This results could also be presented as the risk of introduction of the FMDV into FMD-FZWoutV per year, been in average the probability of 0.0017 per year through bone-in beef (Table 1).

**Table 1:** Results of a quantitative assessment for the annual risk of foot-and-mouth Disease (FMD) virus introduction into the FMD-free zone without vaccination from the FMD-free zone with vaccination of Argentina through trade of bone-in beef and movement of live domestic pigs, and sheep and goats.

	**Result associated to trade of bone-in beef assuming an outbreak in the FMD-FZWV**		**Result associated to introduction of swine**				**Result associated to introduction sheep and goat**	
			**Assuming an outbreak in the FMD-FZWV with a bovine-adapted strain**		**Assuming an outbreak in the FMD-FZWV with a swine-adapted strain**		**Assuming an outbreak in the FMD-FZWV with a bovine-adapted strain**	
	According to the ratio bone-in to deboned beef entering Northern Patagonia A until 2012	Considering illegal trade of bone-in beef	Without serological testing	With serological testing	Without serological testing	With serological testing	Without serological testing	With serological testing
Mean	0.0017	0.000018	**0.08**	0.0075	**0.14**	**0.0148**	0.006	0.0007
CI (95%)	(0.00025, 0.00457)	(0.0000012, 0.0000431)	(0.002, 0.359)	(0.0001, 0.04)	(0.04, 0.597)	(0.0001, 0.083)	(0.0001, 0.0165)	(0.00001, 0.00326)

*Mean values higher than the maximum level of risk acceptable for the country (0.01) are bolded*.

The risk posed by illegal trade (1,8 × 10^−5^ per year) is much lower than that associated with legal trade, because of the larger number of movements expected for the later compared to the former. However, if the number of animals introduced through illegal trade would equal that associated with legal trade, then risk associated with the former would be seven times larger than that associated with the later.

If the hypothesized FMD epidemic in the FMD-FZWV affected swine, in all the scenarios, the risk would be higher than the acceptable value (>0.01), except when a serological test is applied prior to shipping. For the bovine-adapted strain, which is expected to have a relatively low morbidity rate and it is less likely to be detected, if a serological test is not performed, it is expected that an infected pig every 12 epidemics would enter into FMD-FZWoutV (probability of FMDV introduction of 0.08 per year). However, if a serological test was performed, the risk would be reduced to one pig every 130 epidemics, or in other words, the probability of FMDV entering FMD-FZWoutV without being detected is in average 0.0075 per year. For sheep and goat, even without performing the serological test, the risk was always negligible.

The models, for all assessed routes of introduction, were sensitive mainly to the assumed prevalence within and between premises, using Spearman rank correlation (Tables 2, 3).

**Table 2:** Sensitivity analysis results for trade of bone-in beef (Rank correlation values).

	**According the ratio of bone-in beef/deboned beef that entered to Northern Patagonia A until 2012**	**Considering illegal trade of bone-in beef**
Prevalence within premises (P2)	0.89	0.91
Prevalence between premises (P1)	0.38	0.40
Sensitivity of ante-mortem inspection (P4a)	0.13	–
Sensitivity of post-mortem inspection (P5a)	0.12	–

**Table 3:** Sensitivity analysis results for susceptible unvaccinated species (Rank correlation values).

	**Swine**				**Sheep and goat**	
	**Bovine adapted strain**		**Swine adapted strain**		**Bovine-adapted strain**	
	**With serological testing**	**Without serological testing**	**With serological testing**	**Without serological testing**	**With serological testing**	**Without serological testing**
Prevalence within premises (P2)	0.61	0.70	0.61	0.71	0.46	0.75
Prevalence between premises (P1)	0.55	0.64	0.55	0.64	0.38	0.63
Sensitivity of clinical pre-shipping inspection (P3)	0.15	0.19	0.16	0.19	0.05	0.09
Sensitivity of serological test	−0.48	–	−0.48	–	−0.76	–

## Discussion

Results suggest that Argentine regulations may become more flexible, still in agreement with international regulations. The risk associated with removing some of the prevailing restrictions to trade between the FMD-FZWV and the FMD-FZWoutV of Argentina was estimated to be negligible, even when an FMD epidemic was assumed to occur in the former and conservative assumptions were assumed for the assessment. The risk associated to bone-in beef, through legal and illegal trade, is <0.01 per year (0.0017 and 0.000018, respectively).

Because of the negative impact that such restrictions have in the development of the region, results here suggest the convenience of relaxing such restrictions, without further affecting the sanitary status of the region. Such mitigations actions would be consistent with a need for adapting national legislation to the plans outlined at the Hemispheric Program for the Eradication of Foot-and-Mouth Disease, which recommends discontinuing FMD vaccination activities in the Americas.

To estimate the probabilities required for the risk assessment, data were collected from different time frames, in order to use the best data available. Because the sanitary situation related to FMD in Argentina has been stable since 2006, current data are not available and the most accurate option for simulating risk is to use data collected on recent experiences with FMD outbreaks in the country. Also, because the primary intention of the manuscript was to evaluate the appropriateness of the current legislation specifically, other potential routes of spread, such as the infection through fomites or vehicles, fell beyond the scope of the study and were not considered here.

When the volume of legal and illegal trade was similar, then the risk associated with illegal trade was seven times higher compared to legal trade. Illegal trade was also found as a major risk for the introduction of FMD elsewhere (32). These results may be explained, at least in part, because there are no options to perform any ante- or post-mortem inspections of illegally shipped cattle. Consequently, one may state that, given the negligible risk estimated here, the illegal nature of the movements unnecessarily increases the risk of introduction into the region. For that reason, one would argue that the restrictions results on a paradoxical effect, in which the risk is exacerbated by a measure, intended to reduce it. Furthermore, allowing legal trade of beef would discourage the illegal trade; this risk management is expected to decrease the risk, already estimated as negligible. Meat maturation is a rational risk reduction measure if there is a probability of virus being present in the carcass. Furthermore, because vaccination reduces FMD morbidity in cattle and thus decreases the risk of slaughtering viraemic cattle, together, maturation, and vaccination results in high chances of meat free of virus being traded (33). Also, considering the probabilities of carriers, the absence of viraemia in these animals reduces the risk of the meat (34). Therefore, the vaccination program and the monitoring of immunity are the most important warranties to allow the trade of beef, combined with a sensitive early detection surveillance system.

Short ribs are a highly demanded product in Argentina, and such demand is not fully covered by the local production in the FMD-FZWoutV, because most of the livestock production takes place in the FMD-FZWV. Consequently, attempts to smuggling beef or animals through the Patagonic Phytozoosanitary barrier, which are controlled by confiscation in the different checkpoints, are not rare. Because the last FMD outbreak in Patagonia occurred in 1994, including a number of epidemics in the FMD-FZWV, one may argue that those inspections have been a sufficient mitigation measure for the risk of FMDV introduction into the region.

For susceptible and unvaccinated species, specifically for sheep and goat, the risk was always negligible, with a serological test (0.0007 per year) and without it (0.006 per year). The risk was also negligible for swine infected with a bovine-adapted strain subjected to a serological test (0.0075 per year), which, traditionally, have been the most common strains in South America. Performing a serological test prior to movement is important to mitigate risk in case of infected animals without clinical signs, which typically occurs over the silent phase of an epidemic (35). But this mitigation measure may not be enough. That is the case when a swine-adapted strain outbreak is evaluated. The risk is higher than accepted in pigs, applying (0.0148 per year) or not a serological test (0.14 per year) prior movement. For pigs, the relatively high risk was consistent with estimates from a quantitative risk assessment of FMD introduction into Spain via importation of live animals (20), in which the probability of FMDV introduction was estimated to be more likely to occur via importation of live pigs than through importation of other susceptible species. That result may be explained because of the extremely conservative assumption which may have resulted on an overestimation of the prevalence within premises, which was considered four times higher, and between premises, which was considered twice higher if the virus was more adapted to pigs than to cattle.

Results were most sensitive to those two parameters (i.e., within- and between-farm prevalence), further suggesting that the impact of a potential overestimation may have had on the estimates of risk. It is expected that in the unlikely event that a serotype O swine-adapted strain, such as the FMDV that affected Taiwan in 1997, enters into the country, it will be quickly detected because swine are not protected against that antigen, they would develop an evident clinical disease, facilitating its detection and control. However, when swine was affected with the most common strain in the region (i.e., cattle-adapted strains), if animals were tested prior to shipping, the risk was nil. Noteworthy, that would be the most likely situation in the event of an epidemic affecting swine in the country, with infection in swine expected to be evident, given that pigs are not vaccinated in Argentina. For swine-adapted strains, such as O Taiwan 1997, the risk was not acceptable even if a serological test was conducted. However, as explained, the assumptions have been extremely conservative, assuming an epidemic of such strain type in the FMD-FZWV, which has never been reported in Argentina.

The sensitivity analysis was performed to identify the most influential variables in this quantitative model. The results provide an indication of the relative weight of the various input uncertainties and a basis for gathering further information (36). The prevalence of FMDV in the FMD-FZWV between and within premises were the most relevant variables in the sensitivity analysis. Because of the nature of the process, it is difficult to discriminate how much of that variation was due to uncertainty and how much to variability. However, because conservative values were assumed for those parameters, we argue that it is unlikely that the between and within prevalence assumption has affected the conclusions of the results presented here.

Finally, it should be noted that the analysis here has evaluated the annual risk of introduction of FMDV in the FMD-FZWoutV subsequent to the hypothetical scenario of an FMD outbreak in the FMD-FZWV, which, at the time when this manuscript was written in December 2017, has not occurred for more than a decade in the country. Thus, it should not be interpreted as an annual risk that the event will occur in the current situation. Actually, in the current situation, when there is solid epidemiological evidence about the absence of viral circulation and there are not diseased animals, the risk is likely to be lower than the estimates here. Every year SENASA executes an epidemiological surveillance to prove the absence of viral circulation (37). Additionally, all suspected cases of the disease are investigated and, in the last years, all suspects have been dismissed.

In summary, the risk for introduction of FMD into the FMD-FZWoutV of Argentina associated with a relaxation of the restrictions currently imposed to trade is negligible. For those reasons, and because of the negative impact that the measure has in the region, it is recommended the revision of the restrictive policy implemented by the country.

## Author Contributions

AP and AM planned the study and equally contributed to writing the manuscript. The analysis were conducted by AM and supervised by AP.

### Conflict of Interest Statement

The authors declare that the research was conducted in the absence of any commercial or financial relationships that could be construed as a potential conflict of interest.
